# Posterior femoral condylar morphology following mechanical vs. kinematic alignment using a medial‐pivot implant designed for mechanical alignment: A CT‐based comparative study

**DOI:** 10.1002/ksa.70022

**Published:** 2025-09-05

**Authors:** Yohei Ohyama, Yukihide Minoda, Sho Masuda, Ryo Sugama, Hideki Ueyama, Hidetomi Terai

**Affiliations:** ^1^ Department of Orthopaedic Surgery Osaka Rosai Hospital Osaka Japan; ^2^ Department of Orthopaedic Surgery Osaka Metropolitan University Graduate School of Medicine Osaka Japan

**Keywords:** kinematic alignment, mechanical alignment, morphology of the posterior femoral condyles, posterior longitudinal overhang in the femoral condyle, total knee arthroplasty

## Abstract

**Purpose:**

The kinematic alignment (KA) technique aims to restore native joint anatomy; however, the extent to which it restores posterior femoral condylar morphology after total knee arthroplasty (TKA) remains unclear. The posterior longitudinal overhang in the femoral condyle (PLOF) has been reported to affect clinical outcomes. This study aimed to compare the PLOF after medial pivot TKA performed using KA and mechanical alignment (MA) techniques.

**Methods:**

This retrospective computed tomography‐based study included 50 knees (from 25 men and 25 women) with medial knee osteoarthritis who underwent unilateral TKA. Femoral component‐computer‐aided design (FC‐CAD) models of a medial‐pivot prosthesis originally engineered for MA were positioned on preoperative computed tomography images according to KA and MA protocols. The primary outcomes were medial and lateral PLOF, defined as the sagittal overhang of the FC‐CAD model beyond the native bone contour. The extent of distal and posterior femoral resections and correlations among femoral morphological parameters, including femoral valgus, lateral distal femoral, and condylar twist angles, and the PLOF were analysed.

**Results:**

The mean medial and lateral PLOF associated with KA was significantly greater (3.8 ± 1.4 vs. 3.2 ± 2.0 mm; *p* = 0.002) and smaller (3.4 ± 1.6 vs. 4.1 ± 1.9 mm; *p* < 0.001), respectively, than those associated with MA. Compared with KA, MA decreased valgus alignment and added external rotation. The PLOF associated with KA and femoral morphological parameters was not significantly correlated.

**Conclusions:**

The KA resulted in significantly different ( > 3 mm) medial and lateral PLOF compared with the MA. The lack of correlations between the KA‐associated PLOF and femoral morphological parameters suggests a native‐prosthetic morphological mismatch. Surgeons should consider strategic downsizing when an MA‐oriented medial‐pivot FC is implanted using KA or employ KA‐specific implants to optimise clinical outcomes.

**Level of Evidence:**

Level III, retrospective comparative study.

**Abbreviations**
3D: three‐dimensional
CAD: computer‐aided design
CI: confidence interval
CT: computed tomography
CTA: condylar twist angle
FC: femoral component
FVA: femoral valgus angle
ICC: intraclass correlation coefficient
KA: kinematic alignment
LDFA: lateral distal femoral angle
MA: mechanical alignment
PLOF: posterior longitudinal overhang in the femoral condyle
PROMs: patient‐reported outcome measures
SD: standard deviation
TKA: total knee arthroplasty

## INTRODUCTION

Mechanical alignment (MA) has traditionally been prioritised in total knee arthroplasty (TKA) to enhance longevity [15, 21]. However, emphasis on patient satisfaction and patient‐reported outcome measures (PROMs) has introduced kinematic alignment (KA), fostering advances in the study of knee phenotype concepts in the coronal, rotational and sagittal alignments [6, 7, 8, 11, 19, 20, 29]. Some studies have reported no compromise in durability, even with implants positioned outside the target range of MA [1, 32], encouraging the development of modified alignment techniques reflecting native knee anatomy [9, 11, 15, 21]. Nevertheless, the impact of these modified techniques on patient satisfaction, functional outcomes, and implant longevity remains uncertain, necessitating further investigation into implant choice and surgical methods [5, 10, 15, 21, 25].

**Figure 1:**
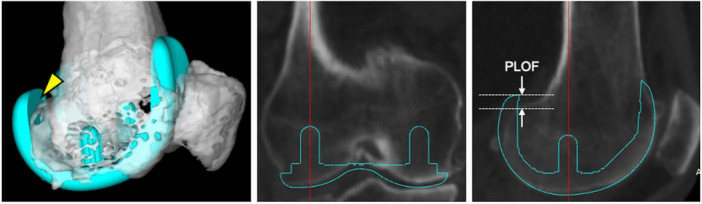
The posterior longitudinal overhang in the femoral condyle. The posterior longitudinal overhang in the femoral condyle (PLOF) is defined as the length of the proximal. sagittal projection of the femoral component (FC) beyond the posterior condylar bone resection surface. along the central axis of the peg (white arrow). The yellow arrowhead in the left image indicates the three dimensional proximal overhang of the posterior femoral condyle in the FC. The central image represents a coronal plane view, while the sagittal plane along the central axis of the peg (red line) is shown in the right image. The distance between the white arrows is measured as the PLOF.

Howell et al. first proposed KA in 2008 using patient‐specific instruments designed to align with the three femoral kinematic axes [11]. In 2019, the 'calipered' KA technique was introduced; it aims to restore native knee anatomy in extension and flexion by using standard instruments and compensating for 2 mm of cartilage wear and sawblade thickness to match the implant thickness within 0.5 mm of the resected bone [9]. While the KA technique restores coronal and rotational anatomy, evidence regarding its sagittal congruence with the femoral component (FC) remains limited. Because native femoral condylar radius and centre depend on individual skeletal parameters [22], the current MA‐oriented FCs may not align with the original femoral curvature, raising concerns over posterior longitudinal overhang in the femoral condyle (PLOF) (Figure 1) [26]. The PLOF has been reliably measured using a three‐dimensional (3D) computed tomography (CT)–computer‐aided (CAD) image‐matching system and associated with reduced knee flexion and worse PROMs [26]. Thus, the PLOF holds theoretical and clinical relevance for the KA technique, highlighting the importance of addressing posterior femoral condyle restoration in the sagittal alignment.

This retrospective CT‐based study selected an MA‐oriented medial‐pivot prosthesis with a single‐radius posterior geometry, designed to approximate native femoral anatomy [4], to isolate how such an implant might mismatch posterior condylar morphology under the KA protocol. This study aimed to compare the PLOF with an MA‐oriented medial pivot FC under KA versus MA and examine its relationship with femoral morphological parameters, sex, or FC size. We hypothesized that, even under the KA protocol, the implantation of an MA‐oriented medial pivot FC would not restore native posterior condylar morphology and would produce a PLOF profile distinct from that of MA.

## MATERIALS AND METHODS

### Study design, patient selection and ethical approval

This 3D imaging analysis was conducted retrospectively in patients with knee osteoarthritis who underwent unilateral TKA at our institution. The cohort included a consecutive series of 36 male (37 knees) from 24 August 2021 to 15 December 2023, and 47 female patients (50 knees) from 14 January 2022 to 10 January 2023. Cases involving bilateral TKA (two and six knees in men and women, respectively), diseases other than medial knee osteoarthritis (e.g., lateral knee osteoarthritis, rheumatoid arthritis, and spontaneous osteonecrosis of the knee; 6 and 14 knees in men and women, respectively), substantial bone defects requiring the use of a mega prosthesis, metal augmentation, or a stem (one and three knee in men and women, respectively), and revision TKA (three and two knees in men and women, respectively) were excluded. Finally, the data of 25 knees with medial osteoarthritis in each sex category were analysed. Patient demographics are presented in Table 1. For each sex category, 13 right and 12 left knees were included.

**Table 1:** Patient demographics and femoral morphological parameters.

Variable	All patients			Male patients			Female patients			*p*‐Value^a^
	Mean	SD	Range	Mean	SD	Range	Mean	SD	Range	
Age (years)	76.1	8.0	46–88	75.5	7.6	58–87	76.8	8.5	46–88	0.565
Height (cm)	156.5	10.0	139–181	162.8	8.5	150–181	150.1	6.8	139–164	<0.001
Weight (kg)	63.1	11.5	43–94	68.4	10.9	51–94	57.9	9.8	43–88	<0.001
BMI (kg/m^2^)	25.7	3.2	10.2–32.7	25.7	3.0	20.2–31.6	25.6	3.5	20.5–32.7	0.903
FVA (°)	5.9	2.3	1.9–13.0	5.6	1.8	1.9–8.4	6.3	2.7	2.5–13.0	0.244
LDFA (°)	87.9	2.6	82.8–94.4	88.4	2.1	83.7–91.7	87.5	3.0	82.8–94.4	0.223
CTA (°)	3.4	1.4	0.7–6.7	2.6	1.1	0.7–4.4	4.3	1.1	1.2–6.7	<0.001

Abbreviations: BMI, body mass index; CTA, condylar twist angle; FVA, femoral valgus angle; LDFA, lateral distal femoral angle; SD, standard deviation

^a^ Comparison between male and female patients: unpaired *t*‐test.

### 3D templating procedure and prosthesis positioning

**Figure 2:**
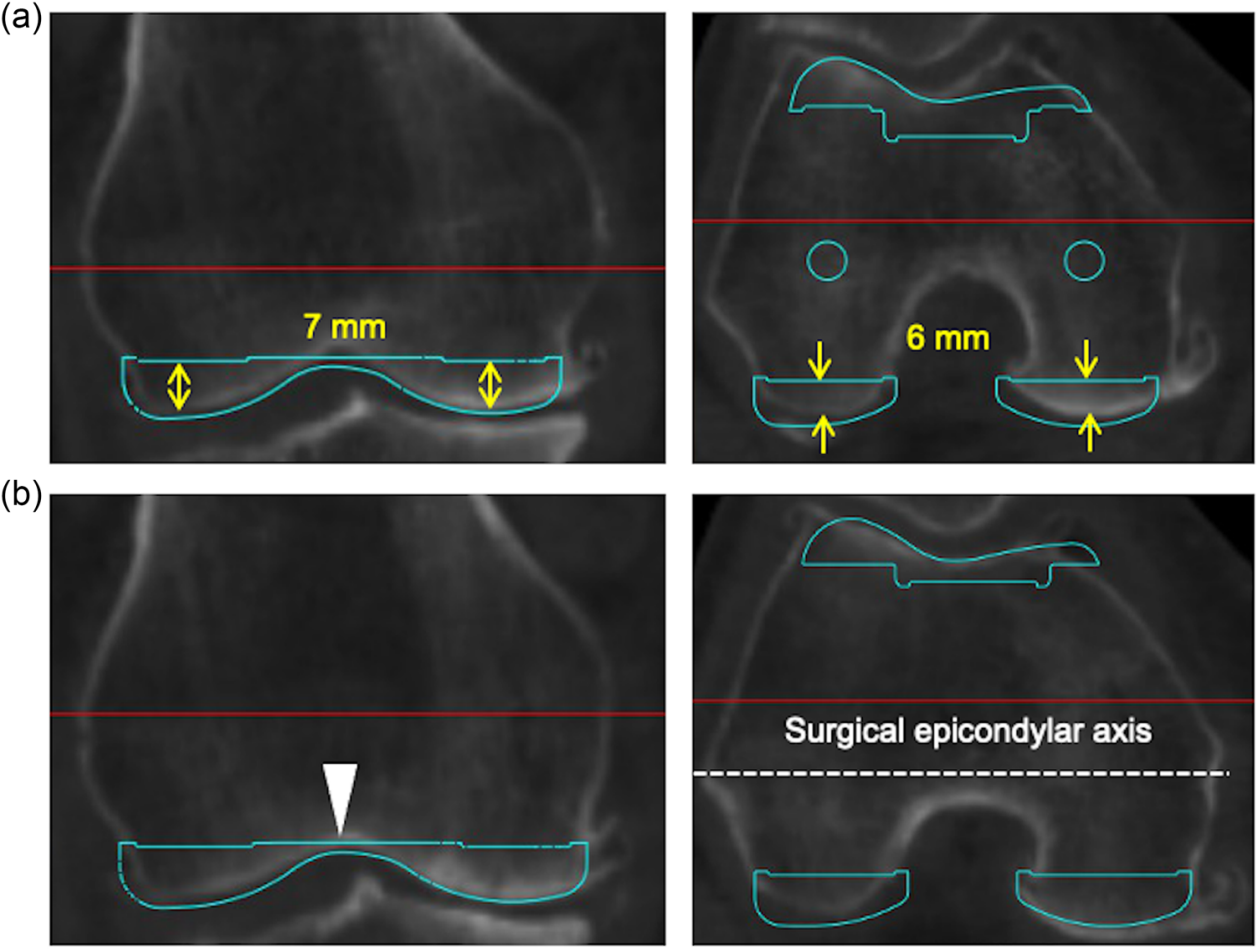
Femoral component placement protocols for kinematic and mechanical alignment techniques. (a) In the kinematic alignment technique, the femoral component (FC) is positioned to achieve 7 mm of distal femoral resection and 6 mm of posterior condylar resection. (b) In the mechanical alignment technique, the FC is positioned such that the distal resection surface. coincides with the deepest point of the trochlear groove (white arrowhead), with the rotation aligned parallel to the surgical epicondylar axis.

Using 3D templating software (ZedKnee, LEXI Co., Ltd., Tokyo, Japan), FC‐CAD models based on a medial pivot prosthesis (GMK Sphere, Medacta International AG, Inc., Castel San Pietro, Switzerland) originally engineered for MA were positioned on the preoperative CT images. The GMK Sphere has been adopted in multiple seminal calipered KA‐TKA studies, including those by Howell et al., which consistently report its femorotibial kinematic patterns and clinical outcomes [2, 3, 13, 23, 31], thereby validating its use for analysis of posterior condylar morphology in our study. The radii of the medial and lateral condyles of the FC were consistent (25 mm for size 1–3+ and 30 mm for size 4–7) with distal and posterior FC thicknesses of 9 and 8 mm, respectively. In KA, the FC‐CAD model was positioned to match the thickness of the implant with 7‐mm distal femoral resection and 6‐mm posterior femoral resection, accounting for 2‐mm cartilage wear (Figure 2a) [9]. After planning 7 mm distal and 6 mm posterior resections, the smallest AP size was chosen to ensure that the anterior cut lay flush with the native anterior femoral cortex without creating an anterior notch [28]. In MA, the FC‐CAD model was positioned according to the sulcus cut technique, aligning it with the deepest point of the trochlear groove [17] and parallel to the surgical epicondylar axis [34]. The FC‐CAD model's size was selected such that the average resection depth of the medial and lateral posterior condyles, corrected for 2‐mm cartilage wear, most closely matched the thickness of the implant (Figure 2b). The FC‐CAD model was flexed at 3° (range 2°–4°) relative to the sagittal mechanical axis of the femur from the centre of the femoral head, reflecting natural anterior bowing [29, 33].

### Outcome measures

The primary outcome was the medial and lateral PLOF, and the alignment techniques were compared. As previously reported, the PLOF was defined as the sagittal overhang of the FC‐CAD model beyond the native bone contour—excluding osteophytes—at the respective central axis of medial and lateral pegs in the implant coordinate system (Figure 1), and was recorded to one decimal place [26]. Sub‐analyses of the PLOF measurements included comparisons between sexes and between FC sizes (small [size 1–3+ with a radius of 25 mm] and large [size 4–7 with a radius of 30 mm]). Secondary outcomes included differences in the extent of medial and lateral distal and posterior femoral resections for each technique. Additionally, correlations of femoral morphological parameters, including the femoral valgus angle (FVA) [16], lateral distal femoral angle (LDFA) [19, 20], and condylar twist angle (CTA) [34] with the PLOF were assessed. The FVA and LDFA were measured using preoperative long‐leg standing anteroposterior radiographs, whereas the CTA was measured using preoperative knee CT images. The FVA was defined as the angle between the mechanical and anatomical axes of the distal femur [16]. The LDFA was the lateral angle formed by the femoral mechanical axis and a line across the articular surface at the distal lateral and medial condyles [19, 20]. The CTA was the angle between the posterior condylar and surgical epicondylar axes [34]. The Institutional Review Board of Osaka Metropolitan University Graduate School of Medicine approved this study before commencement (Approval No. 1280). All participants provided informed consent.

### Statistical analyses

Sample size was calculated using G* Power 3.1.9.4 (Heinrich‐Heine‐Universität Düsseldorf, Düsseldorf, Germany). Based on a previously reported threshold difference of 1.1 mm in lateral PLOF [26] and an estimated standard deviation (SD) of 2.4 mm (pilot data), at least 40 cases were necessary to achieve a power of 0.80 at a significance level (α) of 0.05 using paired *t‐*tests. The normality of the variables was confirmed using the Shapiro–Wilk test. Normally distribution variables are presented as the mean ± SD (range). A paired t‐tests were used for KA–MA comparisons, and an unpaired t‐tests were utilised for sex and FC‐size comparisons. Correlations between the PLOF and femoral parameters were assessed using Pearson correlation coefficients. Two blinded observers (YO and SM) evaluated the inter‐ and intra‐observer reliabilities for the medial and lateral PLOFs using the 3D templating software. This analysis was based on the data from 10 knees (five men and five women) using the interclass correlation coefficient. Measurements were repeated at intervals exceeding 2 weeks. Statistical analyses were conducted using BellCurve for Excel (ver. 4.08; Social Survey Research Information Co., Ltd., Tokyo, Japan), with significance set at *p* < 0.05.

## RESULTS

For medial PLOF, the intra‐observer intraclass correlation coefficient (ICC) was 0.95 (95% confidence interval [CI], 0.80–0.99), and the inter‐observer ICC was 0.95 (95% CI, 0.68–0.99). For lateral PLOF, the intra‐ and inter‐observer ICCs were 0.98 (95% CI, 0.94–1.00) and 0.98 (95% CI, 0.84–1.00), respectively, indicating excellent reliability.

**Figure 3:**
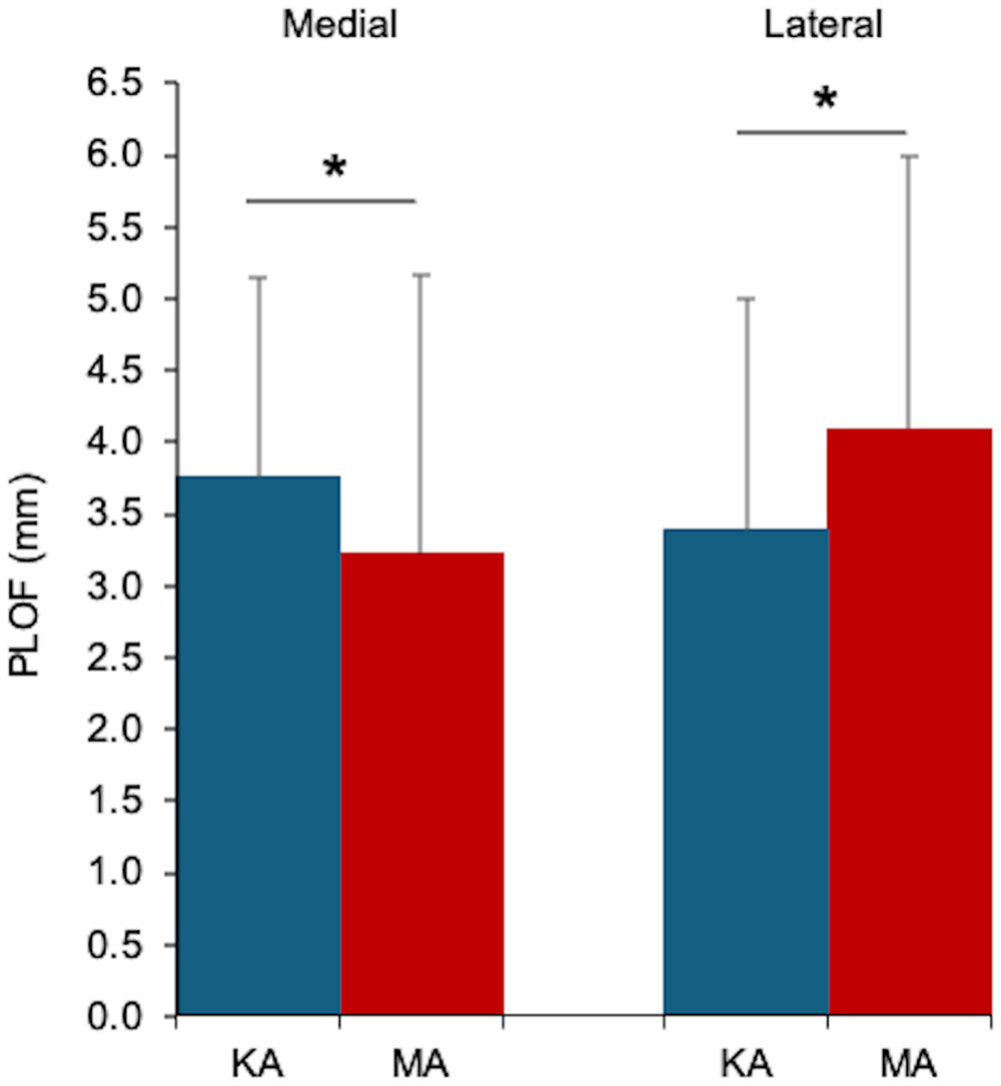
A bar graph comparing medial and lateral PLOF between the KA and MA techniques. The mean medial PLOF was significantly larger with KA than with MA (3.8 ± 1.4 [0.5–7.0] mm vs. 3.2 ± 2.0 [0–8.6] mm; *p* = 0.002). The mean lateral PLOF was significantly smaller with KA than with MA (3.4 ± 1.6 [0–7.1] mm vs. 4.1 ± 1.9 [0–7.5] mm; *p* < 0.001). Bars indicate mean values, and error bars represent standard deviations. *p < 0.05; paired *t‐*test. KA, kinematic alignment; MA, mechanical alignment; PLOF, posterior longitudinal overhang in the femoral condyle.

The PLOF was observed in all knees for both alignment techniques (Table 2 and Figure 3). The mean medial PLOF was significantly larger with KA than with MA (3.8 ± 1.4 mm vs. 3.2 ± 2.0 mm; *p* = 0.002). Conversely, mean lateral PLOF was significantly smaller with KA than with MA (3.4 ± 1.6 mm vs. 4.1 ± 1.9 mm; *p* < 0.001) (Table 2 and Figure 3).

**Table 2:** The posterior longitudinal overhang in the femoral condyle and the extent of bone resection for each alignment technique.

Variable		KA technique			MA technique			*p*‐Value^a^	Difference (KA – MA)		
		Mean	SD	Range	Mean	SD	Range		Mean	SD	Range
PLOF (mm)	Medial	3.8	1.4	0.5–7.0	3.2	2.0	0–8.6	0.002	0.5	1.2	−1.9 to 3.3
	Lateral	3.4	1.6	0–7.1	4.1	1.9	0–7.5	<0.001	−0.7	1.3	−3.6 to 2.2
Extent of resection of the distal femur (mm)	Medial	7.0	NA	NA	7.8	1.3	4.2–10.3	NA	−0.8	1.3	−3.3 to 2.8
	Lateral	7.0	NA	NA	6.1	1.2	4.0–8.6	NA	0.9	1.2	−1.6 to 3.0
	Average	7.0	NA	NA	7.0	0.8	5.5–9.0	NA	0.0	0.8	−2.0 to 1.5
Extent of resection of the posterior femoral condyle (mm)	Medial	6.0	NA	NA	7.4	0.5	6.3–8.1	NA	−1.4	0.5	−2.1 to − 0.3
	Lateral	6.0	NA	NA	4.7	0.5	3.9–5.7	NA	1.3	0.5	0.3–2.1
	Average	6.0	NA	NA	6.0	0.1	6.0–6.2	NA	0.0	0.1	−0.2 to 0.1

Abbreviations: CTA, condylar twist angle; FVA, femoral valgus angle; KA, kinematic alignment; LDFA, lateral distal femoral angle; MA, mechanical alignment; NA, not available; PLOF, posterior longitudinal overhang in the femoral condyle; SD, standard deviation.

^a^ Comparison between KA and MA techniques: paired *t*‐test.

Mean differences in the extent of the distal femoral resection (KA − MA) were −0.8 ± 1.3 mm medially and 0.9 ± 1.2 mm laterally, indicating a less valgus FC orientation in MA. Mean differences in the extent of the posterior femoral condylar resection (KA − MA) were −1.4 ± 0.5 mm medially and 1.3 ± 0.5 mm laterally, indicating an externally rotated FC under MA (Table 2).

No significant KA‐based correlations between the PLOF and the FVA, LDFA, or CTA emerged. Under MA, the medial PLOF showed significant negative correlations with the FVA (*r* = −0.441; *p* = 0.001) and LDFA (*r* = −0.388; *p* = 0.005), whereas the lateral PLOF was positively correlated with the FVA (*r* = 0.350; *p* = 0.013) (Table 3).

**Table 3:** Correlations among medial and lateral posterior longitudinal overhang in the femoral condyle and femoral morphological parameters.

Technique	PLOF	FVA	LDFA	CTA
KA technique	Medial PLOF	−0.205	−0.069	−0.187
	Lateral PLOF	0.188	−0.150	0.214
MA technique	Medial PLOF	−0.441*	−0.388*	−0.162
	Lateral PLOF	0.350*	0.204	0.162

Abbreviations: CTA, condylar twist angle; FVA, femoral valgus angle; KA, kinematic alignment; LDFA, lateral distal femoral angle; MA, mechanical alignment; PLOF, posterior longitudinal overhang in the femoral condyle.

* *p* < 0.05.

**Figure 4:**
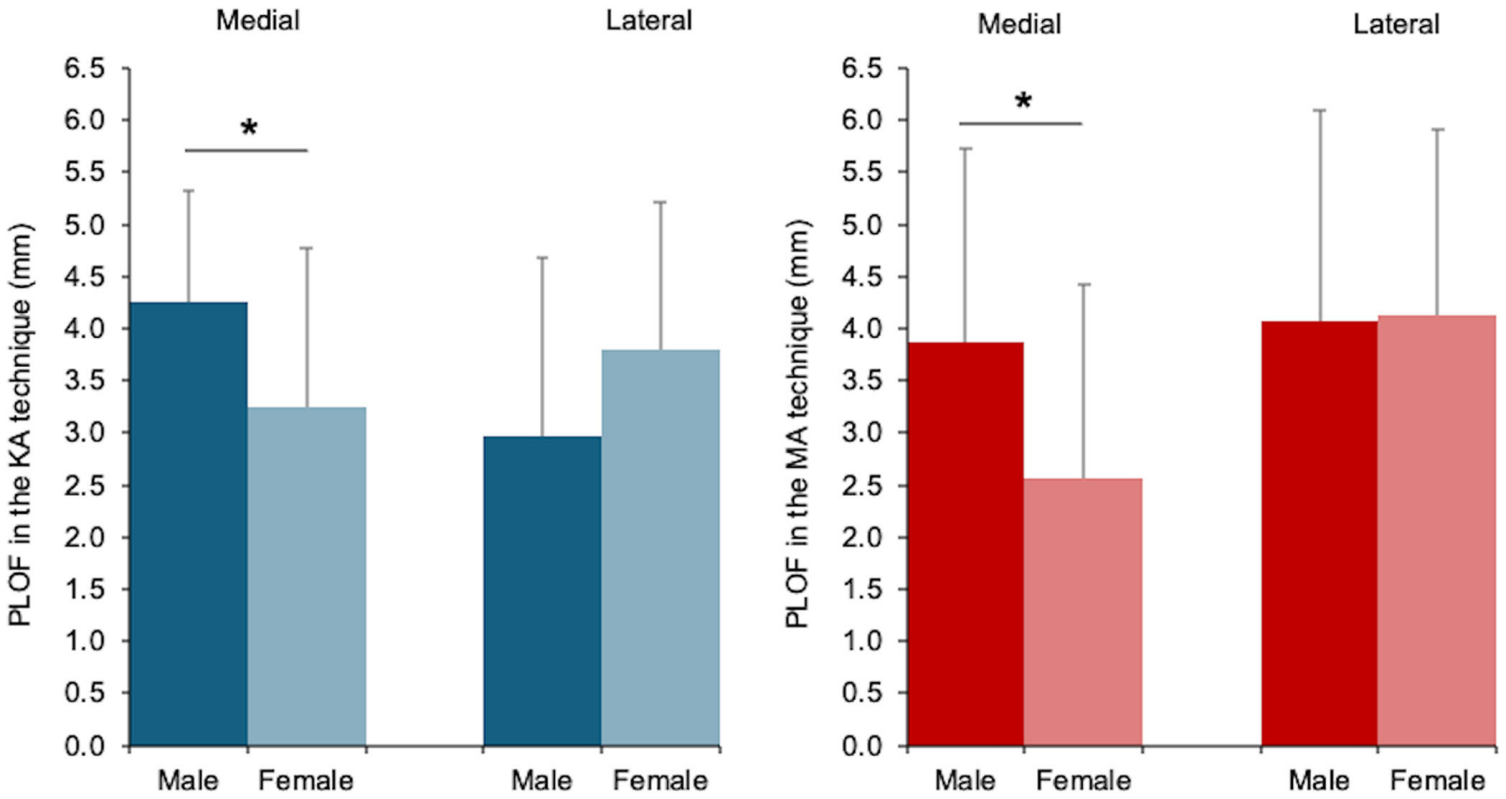
Bar graphs comparing medial and lateral PLOF between men and women for the KA and MA techniques. The medial PLOF was significantly greater in men than in women for both techniques: KA: 4.2 ± 1.1 (3.0–7.0) mm vs. 3.2 ± 1.5 (0.5–5.8) mm; *p* = 0.009 and MA: 3.9 ± 1.9 (1.1–8.6) mm vs. 2.6 ± 1.9 (0–6.3) mm; *p* = 0.016. Neither alignment technique showed significant sex‐based differences in the lateral PLOF. Bars indicate mean values, and error bars represent standard deviations. *p < 0.05; unpaired *t‐*test. KA, kinematic alignment; MA, mechanical alignment; PLOF, posterior longitudinal overhang in the femoral condyle.

**Figure 5:**
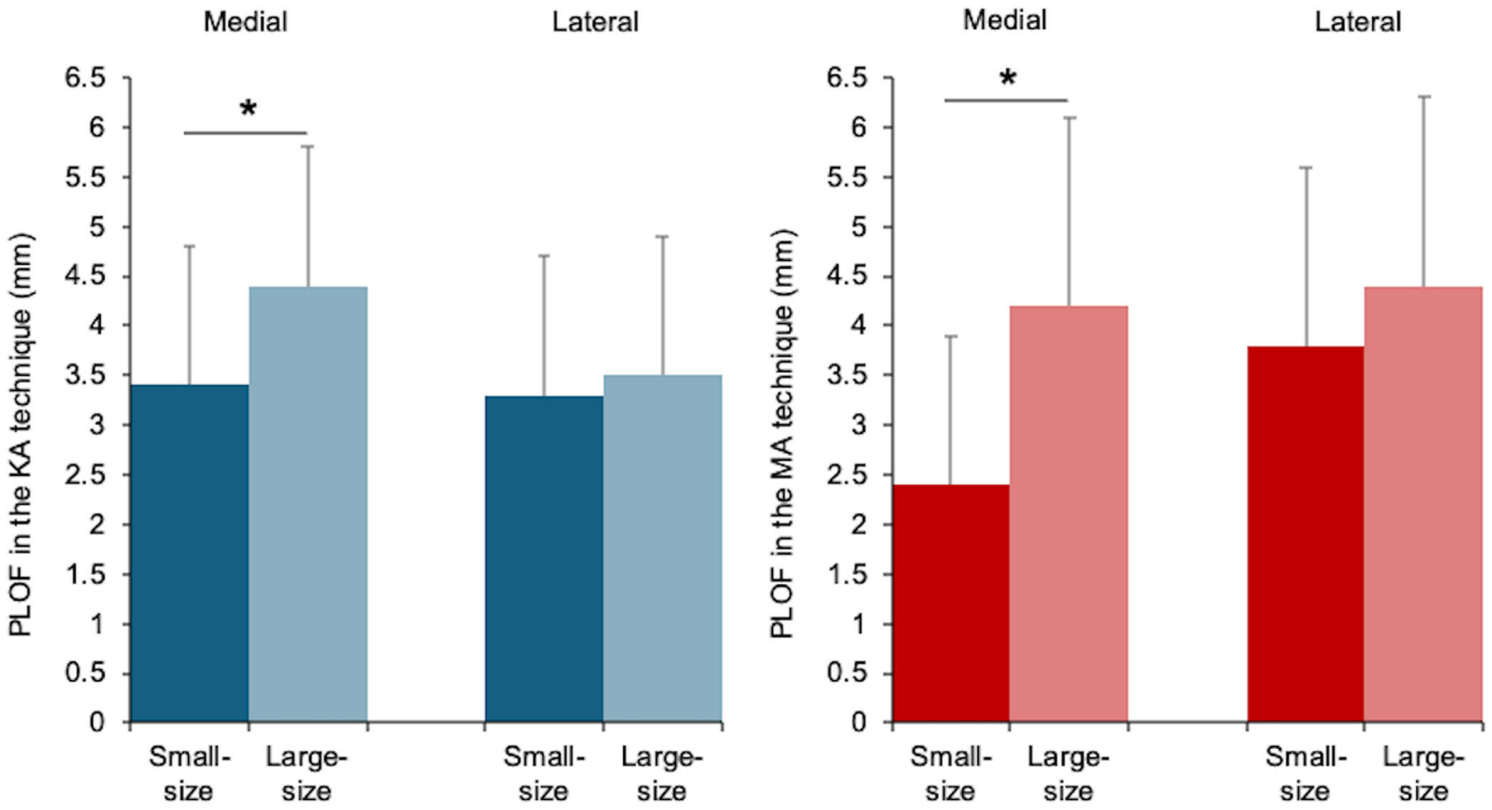
Bar graphs comparing medial and lateral PLOF between small‐ and large‐size femoral component groups for the KA and MA techniques. The medial PLOF with KA was significantly larger in the large‐size group than in the small‐size group (4.4 ± 1.2 [3.0–7.0] mm vs. 3.4 ± 1.4 [0.5–5.8] mm; *p* = 0.011). Similarly, the medial PLOF with MA was larger in the large‐size group than in the small‐size group (4.2 ± 1.9 [1.1–8.6] mm vs. 2.4 ± 1.5 [0–5.1] mm; *p* < 0.001). No significant differences in the lateral PLOF were observed in either alignment technique. Bars indicate mean values, and error bars represent standard deviations. *p < 0.05; unpaired *t‐*test. KA, kinematic alignment; MA, mechanical alignment; PLOF, posterior longitudinal overhang in the femoral condyle.

Neither alignment technique exhibited significant sex‐based differences in the lateral PLOF. The medial PLOF was significantly greater in men than in women for both techniques (KA; *p* = 0.009, MA; *p* = 0.016) (Table 4 and Figure 4). Nine of the 50 knees had a one‐size larger FC with MA than with KA. Among these, five knees had changes in size that altered the posterior condylar radius (e.g., size 3 + –4). Size‐based comparisons showed no significant differences in the lateral PLOF for both alignment techniques. The medial PLOF with KA was significantly larger in the large‐size group than in the small‐size group (*p* = 0.011). Similarly, the medial PLOF with MA was larger in the large‐size group than in the small‐size group (*p* < 0.001) (Table 5 and Figure 5).

**Table 4:** The posterior longitudinal overhang in the femoral condyle with each alignment technique in male and female patients.

Technique	PLOF	Male patients			Female patients			*p*‐Value^a^
		Mean	SD	Range	Mean	SD	Range	
KA technique	Medial PLOF	4.2	1.1	3.0–7.0	3.2	1.5	0.5–5.8	0.009
	Lateral PLOF	3.0	1.7	0–6.1	3.8	1.4	0–7.1	0.065
MA technique	Medial PLOF	3.9	1.9	1.1–8.6	2.6	1.9	0–6.3	0.016
	Lateral PLOF	4.1	2.0	0–7.5	4.1	1.8	0–7.1	0.924

Abbreviations: KA, kinematic alignment; MA, mechanical alignment; PLOF, posterior longitudinal overhang in the femoral condyle; SD, standard deviation

^a^ Comparison between male and female patients: unpaired *t*‐test

**Table 5:** The posterior longitudinal overhang in the femoral condyle with each alignment technique for small and large femoral components.

Technique	PLOF	Small‐size (1–3+ ) (*n* = 32)			Large‐size (4–7) (*n* = 18)			*p*‐Value^a^
		Mean	SD	Range	Mean	SD	Range	
KA technique	Medial PLOF	3.4	1.4	0.5–5.8	4.4	1.2	3.0–7.0	0.011
	Lateral PLOF	3.3	1.5	0–6.1	3.5	1.9	0–7.1	0.628
Technique	PLOF	Small‐size (1–3+) (*n* = 27)			Large‐size (4–7) (*n* = 23)			*p*‐Value*
		Mean	SD	Range	Mean	SD	Range	
MA technique	Medial PLOF	2.4	1.5	0–5.1	4.2	1.9	1.1–8.6	<0.001
	Lateral PLOF	3.8	1.8	0–7.1	4.4	1.9	0–7.5	0.241

Abbreviations: KA, kinematic alignment; MA, mechanical alignment; PLOF, posterior longitudinal overhang in the femoral condyle; SD, standard deviation.

^a^ Comparison between femoral component sizes: unpaired *t*‐test.

## DISCUSSION

This study demonstrated that medial PLOF was significantly greater with KA than with MA, whereas lateral PLOF was significantly smaller with KA than with MA, with both mean PLOF values exceeding 3 mm. Furthermore, under KA, neither medial nor lateral PLOF showed significant correlations with femoral morphological parameters.

Even with KA, an average lateral PLOF of 3.4 mm persisted, although it was significantly lower than that observed with MA. A previous study on medial pivot TKA under MA identified lateral PLOF as a negative predictor for postoperative knee flexion, citing 1.1 mm as a threshold for flexion decrease from baseline [26]. Furthermore, patients showing reduced flexion had worse PROMs for knee stiffness [26]. A larger PLOF effectively shifts the FC posterior condyle beyond the native spacing in deep flexion, elevating joint gap tension and potentially impeding rollback motion [26]. A computer simulation using a cruciate‐substituting TKA with a ball‐and‐socket joint demonstrated that excessive flexion of the FC increased PLOF, causing paradoxical anterior translation of the medial compartment in mid‐flexion [24]. Downsizing the FC (i.e., increasing posterior femoral resection) reduced PLOF and prevented this abnormal kinematics [24]. Moreover, elevated joint lines, caused by increased distal femoral resection, contribute to larger PLOF [26]. A retrieval study has correlated joint line elevation with increased polyethylene wear, suggesting potential long‐term implications [27]. Additionally, a systematic review has shown negative effects of joint‐line elevation on patient function [18]. Our results further show that, compared with KA, MA induced varus alignment and external rotation of the FC, which decreased medial PLOF and increased lateral PLOF. This finding indicates that the extent of posterior condylar resection has a greater influence on PLOF than that of distal resection. Therefore, in KA‐TKA preoperative planning, when PLOF is anticipated, surgeons may optimise clinical outcomes and PROMs by strategically downsizing to minimise PLOF without compromising the anatomical restoration central to KA.

In the MA technique, increasing FVAs and LDFAs inevitably leads to reduced medial distal resection and increased lateral distal resection [16, 19, 20]. Consequently, these parameters revealed negative and positive correlations with medial and lateral PLOF, respectively. Conversely, under KA, the PLOF did not correlate with femoral morphological parameters in the coronal or transverse plane. Therefore, the PLOF under KA may be attributable to a mismatch in design between the native femoral posterior condyle and the posterior condyle of the FC, suggesting a potential need for FC design modifications. Howell et al. have already developed a KA‐specific medial‐pivot TKA with a modified trochlear groove to mitigate potential patellofemoral complications [12, 14, 30]. One important contribution of our study is that it highlights geometric issues beyond simple trochlear restoration when an MA‐oriented implant is used for KA. Therefore, future research should evaluate posterior condylar morphology restoration using the new KA‐specific implant.

The medial pivot FC design incorporates a single‐radius concept based on morphological studies [4]. However, morphological mismatches can produce the PLOF. Recreating the posterior femoral condylar morphology requires careful consideration of the centre, radius, and angle of the arc. While the femoral condyles can be geometrically approximated to a circle, each condyle possesses two distinct centres corresponding to the extension (anterior or inferior) and flexion (posterior) facets [4, 22]. These geometric studies have consistently shown that the lateral condyle can be approximated to a single circle because of the minimal variation in the two centre positions. Conversely, the medial condyle is accurately described by two distinct circles because of the increased relative separation between the centres of curvature [4, 22]. Consequently, despite efforts to balance resection for extension and flexion surfaces in KA, the inherent difference in the centres of the medial femoral condyle renders the accurate replication of the native shape difficult. This discrepancy might have contributed to the sex‐ and size‐related differences in medial PLOF observed in this study. These PLOF differences further support the finding that implant geometric mismatch, rather than alignment technique, is the primary driving factor.

This study had some limitations. First, condylar radii, distances, and angles differ across implants. Many modified alignment methods besides calipered KA exist, and MA‐TKA may be performed using alternative protocols [15, 21]. Surgeons frequently adjust distal and posterior resections intraoperatively, particularly in MA‐TKA, which can reduce PLOF. For example, a prior MA‐based gap balancing study used roughly 1.8 mm and 1.6 mm more resection medially and laterally, respectively, lowering PLOF by approximately 2 mm [26]. Conversely, in KA‐TKA, which aims to restore native morphology, the PLOF may be influenced by discrepancies between the implant design and the patient's native anatomy. However, each FC model possesses unique posterior condylar morphology relative to its anteroposterior size, and only a single MA‐oriented medial pivot FC (not optimised for KA) was evaluated. Therefore, these results may not be generalized to other FC designs. Future studies should investigate the PLOF associated with other prosthesis models, including KA‐specific FCs [14, 30]. Second, implant sizing and placement, determined by a single evaluator, significantly influence PLOF. Nevertheless, consistent criteria were applied for anteroposterior sizing and placement, and it remains important to demonstrate that strategic downsizing may improve outcomes under both MA and KA. Third, the PLOF quantifies two‐dimensional sagittal disparities without accounting for 3D coverage differences. The PLOF has shown clinical significance; [26] however, future research may need to focus on quantifying volumetric assessments. Lastly, this study only enroled patients with medial knee osteoarthritis; the findings may not apply to other conditions.

## CONCLUSIONS

In KA‐TKA using an MA‐oriented medial pivot FC, medial PLOF increased significantly and lateral PLOF decreased significantly compared with MA‐TKA, with both exhibiting a PLOF of >3 mm. Furthermore, the absence of correlations between the PLOF under KA and femoral morphological parameters suggests a design mismatch between the native and prosthetic posterior femoral condyles. Surgeons should employ preoperative simulation to select the implant size that minimises PLOF or use KA‐specific FCs with native posterior condylar radii to optimise postoperative clinical outcomes and PROMs in KA‐TKA.

## AUTHOR CONTRIBUTIONS

**Yohei Ohyama**: Conceptualisation; methodology; investigation; data curation; formal analysis; writing–original draft preparation; writing–review and editing. **Yukihide Minoda**: Conceptualisation; methodology; project administration; supervision; investigation; data curation; writing–review and editing. **Sho Masuda**: Investigation; data curation; writing–review and editing. **Ryo Sugama**: Investigation; data curation; writing–review and editing. **Hideki Ueyama**: Investigation; data curation; writing–review and editing. **Hidetomi Terai**: Project administration; supervision; writing–review and editing.

## CONFLICT OF INTEREST STATEMENT

The authors declare no conflict of interest.

## ETHICS STATEMENT

The study was approved by the Institutional Review Board in Osaka Metropolitan University Graduate School of Medicine (IRB No. 1280) prior to commencement. Informed consent was obtained from all individual participants included in the study.

## Data Availability

The data that support the findings of this study are available on request from the corresponding author. The data are not publicly available due to privacy or ethical restrictions.

## References

[1] Abdel MP , Ollivier M , Parratte S , Trousdale RT , Berry DJ , Pagnano MW . Effect of postoperative mechanical axis alignment on survival and functional outcomes of modern total knee arthroplasties with cement: a concise follow‐up at 20 years. J Bone Jt Surg. 2018;100:472–478. 10.2106/JBJS.16.01587 29557863

[2] Elorza SP , O'Donnell E , Nedopil A , Howell SM , Hull ML . Ball‐in‐socket medial conformity with posterior cruciate ligament retention neither limits internal tibial rotation and knee flexion nor lowers clinical outcome scores after unrestricted kinematically aligned total knee arthroplasty. Int Orthop. 2023;47:1737–1746. 37195465 10.1007/s00264-023-05834-6

[3] Ettinger M , Tuecking LR , Savov P , Windhagen H . Higher satisfaction and function scores in restricted kinematic alignment versus mechanical alignment with medial pivot design total knee arthroplasty: a prospective randomised controlled trial. Knee Surg Sports Traumatol Arthrosc. 2024;32:1275–1286. 38501253 10.1002/ksa.12143

[4] Freeman MAR , Pinskerova V . The movement of the normal tibio‐femoral joint. J Biomech. 2005;38:197–208. 15598446 10.1016/j.jbiomech.2004.02.006

[5] Gibbons JP , Zeng N , Bayan A , Walker ML , Farrington B , Young SW . No difference in 10‐year clinical or radiographic outcomes between kinematic and mechanical alignment in TKA: a randomized trial. Clin Orthop Relat Resh. 2025;483:140–149. 10.1097/CORR.0000000000003193 PMC11658733 39145997

[6] Hess S , Chelli S , Leclercq V , Lustig S , Graichen H , Hirschmann MT . Three‐compartment phenotype concept of total knee arthroplasty alignment: mismatch between distal femoral, posterior femoral, and tibial joint lines. J Arthroplasty. 2025;40(8):2023–2034. 40049560 10.1016/j.arth.2025.02.015

[7] Hirschmann MT , von Eisenhart‐Rothe R , Graichen H , Vendittoli PA , Chen AF , Lustig S , et al. Neutrality, normality, abnormality and pathology in coronal knee alignment: why and how should we define it in the era of personalised medicine? Knee Surg Sports Traumatol Arthrosc. 2024;32:515–517. 38415940 10.1002/ksa.12107

[8] Hirschmann MT , Khan ZA , Sava MP , von Eisenhart‐Rothe R , Graichen H , Vendittoli PA , et al. Definition of normal, neutral, deviant and aberrant coronal knee alignment for total knee arthroplasty. Knee Surg Sports Traumatol Arthrosc. 2024;32:473–489. 38293728 10.1002/ksa.12066

[9] Howell SM . Calipered kinematically aligned total knee arthroplasty: an accurate technique that improves patient outcomes and implant survival. Orthopedics. 2019;42:126–135. 31099877 10.3928/01477447-20190424-02

[10] Howell SM , Akhtar M , Nedopil AJ , Hull ML . Reoperation, implant survival, and clinical outcome after kinematically aligned total knee arthroplasty: a concise clinical follow‐up at 16 years. J Arthroplasty. 2024;39:695–700. 37659680 10.1016/j.arth.2023.08.080

[11] Howell SM , Kuznik K , Hull ML , Siston RA . Results of an initial experience with custom‐fit positioning total knee arthroplasty in a series of 48 patients. Orthopedics. 2008;31:857–863. 18814593 10.3928/01477447-20080901-15

[12] Howell SM , Sappey‐Marinier E , Niesen AE , Nedopil AJ , Hull ML . Better forgotten joint scores when the angle of the prosthetic trochlea is lateral to the quadriceps vector in kinematically aligned total knee arthroplasty. Knee Surg Sports Traumatol Arthrosc. 2023;31:5438–5445. 37792084 10.1007/s00167-023-07598-3

[13] Howell SM , Shelton TJ , Gill M , Hull ML . A cruciate‐retaining implant can treat both knees of most windswept deformities when performed with calipered kinematically aligned TKA. Knee Surg Sports Traumatol Arthrosc. 2021;29:437–445. 32239272 10.1007/s00167-020-05968-9

[14] Hull ML , Simileysky A , Howell SM . Differences in trochlear morphology of a new femoral component designed for kinematic alignment from a mechanical alignment design. Bioengineering. 2024;11:62. 38247939 10.3390/bioengineering11010062 PMC10812931

[15] Karasavvidis T , Pagan Moldenhauer CA , Haddad FS , Hirschmann MT , Pagnano MW , Vigdorchik JM . Current concepts in alignment in total knee arthroplasty. J Arthroplasty. 2023;38:S29–S37. 10.1016/j.arth.2023.01.060 36773657

[16] Kim JM , Hong SH , Kim JM , Lee BS , Kim DE , Kim KA , et al. Femoral shaft bowing in the coronal plane has more significant effect on the coronal alignment of TKA than proximal or distal variations of femoral shape. Knee Surg Sports Traumatol Arthrosc. 2015;23:1936–1942. 24760162 10.1007/s00167-014-3006-5

[17] Kuriyama S , Hyakuna K , Inoue S , Tanaka Y , Tamaki Y , Ito H , et al. Is a “sulcus cut” technique effective for determining the level of distal femoral resection in total knee arthroplasty? Knee Surg Sports Traumatol Arthrosc. 2014;22:3060–3066. 25100488 10.1007/s00167-014-3217-9

[18] van Lieshout WAM , Valkering KP , Koenraadt KLM , van Etten‐Jamaludin FS , Kerkhoffs GMMJ , van Geenen RCI . The negative effect of joint line elevation after total knee arthroplasty on outcome. Knee Surg Sports Traumatol Arthrosc. 2019;27:1477–1486. 30109369 10.1007/s00167-018-5099-8 PMC6527530

[19] Macdessi SJ , Griffiths‐Jones W , Harris IA , Bellemans J , Chen DB . Coronal Plane Alignment of the Knee (CPAK) classification a new system for describing knee phenotypes. Bone Joint J. 2021;103:329–337. 33517740 10.1302/0301-620X.103B2.BJJ-2020-1050.R1 PMC7954147

[20] Macdessi SJ , Griffiths‐Jones W , Harris IA , Bellemans J , Chen DB . The arithmetic HKA (aHKA) predicts the constitutional alignment of the arthritic knee compared to the normal contralateral knee: a matched‐pairs radiographic study. Bone Joint Open. 2020;1:339–345. 33215122 10.1302/2633-1462.17.BJO-2020-0037.R1 PMC7659698

[21] Macdessi SJ , Oussedik S , Abdel MP , Victor J , Pagnano MW , Haddad FS . The language of knee alignment updated definitions and considerations for reporting outcomes in total knee arthroplasty. Bone Joint J. 2023;105:102–108. 36722056 10.1302/0301-620X.105B2.BJJ-2022-1345

[22] Monk AP , Choji K , O'Connor JJ , Goodfellow JW , Murray DW . The shape of the distal femur: a geometrical study using MRI. Bone Joint J. 2014;96–B:1623–1630. 10.1302/0301-620X.96B12.33964 25452364

[23] Nedopil AJ , Howell SM , Hull ML . More passive internal tibial rotation with posterior cruciate ligament retention than with excision in a medial pivot TKA implanted with unrestricted caliper verified kinematic alignment. Knee Surg Sports Traumatol Arthrosc. 2023;31:852–860. 34921630 10.1007/s00167-021-06840-0 PMC9958185

[24] Nishitani K , Kuriyama S , Nakamura S , Song YD , Morita Y , Ito H , et al. Excessive flexed position of the femoral component causes abnormal kinematics and joint contact/ligament forces in total knee arthroplasty. Sci Rep. 2023;13:6356. 37076503 10.1038/s41598-023-33183-2 PMC10115888

[25] Nucci N , Chakrabarti M , Devries Z , Ekhtiari S , Tomescu S , Mundi R . Kinematic alignment does not result in clinically important improvements after TKA compared with mechanical alignment: a meta‐analysis of randomized trials. Clin Orthop Relat Res. 2025;483:1020–1030. 39842026 10.1097/CORR.0000000000003356 PMC12106242

[26] Ohyama Y , Kobayashi A , Minoda Y , Iwakiri K , Masuda S , Ohta Y , et al. Association between overhang of the posterior femoral condyle and restricted postoperative knee flexion related to patient‐reported stiffness in medial‐pivot total knee arthroplasty. J Arthroplasty. 2025;40:651–657.e2. 39265813 10.1016/j.arth.2024.08.058

[27] Pourzal R , Cip J , Rad E , Laurent MP , Berger RA , Jacobs JJ , et al. Joint line elevation and tibial slope are associated with increased polyethylene wear in cruciate‐retaining total knee replacement. J Orthop Res. 2020;38:1596–1606. 32374428 10.1002/jor.24710 PMC7329363

[28] Rivière C , Dhaif F , Shah H , Ali A , Auvinet E , Aframian A , et al. Kinematic alignment of current TKA implants does not restore the native trochlear anatomy. Orthop Traumatol: Surg Res. 2018;104:983–995. 29960090 10.1016/j.otsr.2018.05.010

[29] Sadoghi P , Hirschmann MT , Karlsson J , Klasan A . The neglected factor of constitutional sagittal alignment and its implications for total knee arthroplasty. Knee Surg Sports Traumatol Arthrosc. 2024;32:10–12. 38226765 10.1002/ksa.12013

[30] Sappey‐Marinier E , Howell SM , Nedopil AJ , Hull ML . The trochlear groove of a femoral component designed for kinematic alignment is lateral to the quadriceps line of force and better laterally covers the anterior femoral resection than a mechanical alignment design. J Pers Med. 2022;12:1724. 36294863 10.3390/jpm12101724 PMC9605321

[31] Sosio C , Rossi N , Sirtori P , Ciliberto R , Lombardo MDM , Peretti GM , et al. Clinical and functional outcomes of kinematic aligned total knee arthroplasty with a medial pivot design: two‐year follow‐up. J Clin Med. 2023;12:7258. 38068313 10.3390/jcm12237258 PMC10707284

[32] Tibbo ME , Limberg AK , Perry KI , Pagnano MW , Stuart MJ , Hanssen AD , et al. Effect of coronal alignment on 10‐year survivorship of a single contemporary total knee arthroplasty. J Clin Med. 2021;10:142. 33406614 10.3390/jcm10010142 PMC7795414

[33] Ueyama H , Minoda Y , Sugama R , Ohta Y , Yamamura K , Nakamura S , et al. Two‐dimensional measurement misidentifies alignment outliers in total knee arthroplasty: a comparison of two‐ and three‐dimensional measurements. Knee Surg Sports Traumatol Arthrosc. 2019;27:1497–1503. 30284009 10.1007/s00167-018-5175-0

[34] Victor J , Van Doninck D , Labey L , Van Glabbeek F , Parizel P , Bellemans J . A common reference frame for describing rotation of the distal femur: a CT‐based kinematic study using cadavers. J Bone Joint Surg Br. 2009;91–B:683–690. 10.1302/0301-620X.91B5.21827 19407308

